# Distribution and Co-Occurrence of Selected Virulence-Associated Genes in *Escherichia coli* Isolates from Urban and Exhibition Pigeons

**DOI:** 10.3390/pathogens15090882

**Published:** 2026-08-22

**Authors:** Alexandru Gligor, Ionica Iancu, Vlad Iorgoni, Paula Nistor, Ionela Popa, Mirela Imre, Mihai Mitulețu, Roxana Popescu, Daliborca Vlad, Andrei Păunescu, Miroslav Urosevic, Emil Tîrziu, Viorel Herman, Ileana Nichita, Agatha Popescu

**Affiliations:** 1 Faculty of Veterinary Medicine, University of Life Sciences “King Mihai I” from Timișoara, 300645 Timișoara, Romania; alexandru.gligor@usvt.ro (A.G.); vlad.iorgoni@usvt.ro (V.I.); paula.nistor@usvt.ro (P.N.); ionela.popa@usvt.ro (I.P.); mirela.imre@usvt.ro (M.I.); emil.tirziu@usvt.ro (E.T.); viorel.herman@fmvt.ro (V.H.); ileana.nichita@usvt.ro (I.N.); 2 Academy of Romanian Scientists, Str. Ilfov No. 3, Sector 5, 050044 Bucharest, Romania; agatha.popescu@aosr.ro; 3 Department of Cellular and Molecular Biology, “Victor Babeș” University of Medicine and Pharmacy, 2 Eftimie Murgu Square, 300041 Timișoara, Romania; roxana.popescu@umft.ro (R.P.); daliborca.vlad@umft.ro (D.V.); 4 ANAPATMOL Research Centre, “Victor Babeș” University of Medicine and Pharmacy, 2 Eftimie Murgu Square, 300041 Timișoara, Romania; 5 Department of Urology, “Pius Brînzeu” Emergency Clinical County Hospital, 300723 Timișoara, Romania; andrei.paunescu@umft.ro; 6 Department of Animal Science, Faculty of Agriculture, University of Novi Sad, Trg Dositeja Obradovića 8, 21000 Novi Sad, Serbia; miroslav.urosevic@polj.uns.ac.rs

**Keywords:** *Escherichia coli*, pigeons, virulence-associated genes, co-occurrence, urban wildlife, exhibition pigeons, One Health

## Abstract

*Escherichia coli* is a highly diverse bacterial species that includes strains with significant pathogenic potential for both animals and humans. Urban pigeons (*Columba livia domestica*) are widely distributed and frequently interact with human environments. In contrast, exhibition pigeons are maintained under controlled conditions, providing an opportunity to compare bacterial populations across different ecological settings. Comparative data on virulence-associated gene patterns between free-living urban and exhibition pigeon populations remain limited, particularly when both populations are investigated within the same geographical setting. The present study aimed to investigate the distribution and co-occurrence patterns of selected virulence-associated genes in *Escherichia coli* isolates recovered from urban and exhibition pigeons. A total of 453 faecal samples (153 urban and 300 exhibition pigeons) were collected, from which 133 *Escherichia coli* isolates were recovered (65 from urban and 68 from exhibition pigeons), corresponding to an overall isolation rate of 29.4%. A representative subset of isolates was independently confirmed by species-specific PCR and Sanger sequencing before virulence gene profiling. Subsequently, all isolates were screened for six virulence-associated genes (*fimC*, *ompA*, *tsh*, *hlyA*, *astA*, and *iroN*) by PCR. Virulence gene prevalence, gene profiles, co-occurrence patterns, and virulence scores were analysed, and statistical comparisons between groups were performed. The most prevalent gene was *ompA* (88.0%), followed by *fimC* (66.2%) and *iroN* (32.3%), whereas *hlyA* was detected only sporadically (3.8%). The most common virulence profile was *fimC + ompA* (24.8%), followed by *ompA* alone (20.3%). Co-occurrence analysis identified *fimC* and *ompA* as the most frequently co-detected gene pair (79/133 isolates; 59.4%); however, their association was weak and not statistically significant (φ = 0.078, *p* = 0.371). Frequent co-detection with the iron acquisition-associated gene *iroN* was also observed. No statistically significant differences were observed in the distribution of the investigated virulence-associated genes between urban and exhibition pigeon isolates, although urban isolates exhibited a broader diversity of gene combinations. In conclusion, pigeon-derived *E. coli* isolates harboured diverse combinations of selected virulence-associated genes, with adhesion-associated genes being the most frequently detected determinants. These findings provide baseline data on the distribution of selected virulence-associated genes in pigeon-derived *Escherichia coli* isolates and support future studies incorporating additional virulence markers, whole-genome sequencing, and comparative analyses to understand their epidemiological relevance better.

## 1. Introduction

*Escherichia coli* is a highly diverse bacterial species that constitutes a normal component of the gastrointestinal microbiota of humans and animals, including birds. While most strains are commensal, certain lineages, collectively referred to as extraintestinal pathogenic *Escherichia coli* (ExPEC), possess a wide range of virulence-associated genes that enable them to cause infections outside the intestinal tract, including urinary tract infections, septicemia, and meningitis [1,2,3]. These strains represent a major public health concern due to their pathogenic potential [1,4,5].

Although *Escherichia coli* is predominantly a commensal organism, ExPEC strains are characterized by the presence of multiple virulence-associated determinants, including adhesins, outer membrane proteins, toxins, and iron acquisition systems, which contribute to their ability to colonize, persist, and cause disease in extraintestinal sites [6,7,8]. Comparative studies have demonstrated significant differences in virulence gene content between commensal and pathogenic strains, highlighting the remarkable genetic plasticity and adaptability of this species [6,7].

Genes such as *fimC*, involved in fimbrial assembly and bacterial adhesion; *ompA,* encoding an outer membrane protein associated with host interaction and serum resistance; *tsh*, a temperature-sensitive hemagglutinin; *hlyA*, encoding hemolysin; *astA*, associated with toxin production; and *iroN*, involved in siderophore-mediated iron acquisition, are frequently investigated as markers of virulence-associated potential.

These six genes were selected to represent different categories of virulence-associated determinants, including adhesion and fimbrial assembly (*fimC*), outer membrane-associated host interaction (*ompA*), autotransporter-mediated adhesion (*tsh*), toxin-associated determinants (*hlyA* and *astA*), and iron acquisition (*iroN*). The selected panel was intended to provide a focused assessment of representative virulence-associated traits previously investigated in avian *E. coli*, rather than to establish ExPEC/APEC classification or to characterise the complete virulome.

Animal and environmental reservoirs play a crucial role in the maintenance and dissemination of ExPEC strains and their associated virulence determinants. Avian species, livestock, and companion animals have been identified as important reservoirs, supporting the concept of interspecies transmission within a One Health framework [9,10,11,12,13].

Several studies have demonstrated that pigeons harbour *Escherichia coli* strains carrying virulence-associated genes, highlighting their importance as a source of genetic diversity within avian-associated bacterial populations [14,15,16,17].

However, comparative information on the distribution and co-occurrence of virulence-associated genes in *E. coli* isolates from free-living urban and exhibition pigeons remains limited, particularly when both populations are investigated within the same geographical setting. Such comparisons may provide baseline information on whether different pigeon populations exhibit distinct patterns of selected virulence-associated genes.

Pigeons (*Columba livia domestica*) are among the most widespread synanthropic bird species and are commonly found in urban environments, where they live in proximity to humans. Their ecological adaptability and continuous exposure to anthropogenic environments, waste, and diverse microbial communities may facilitate their carriage of bacterial strains harbouring virulence-associated determinants [9,10,11,12,18,19,20,21]. In contrast, exhibition pigeons are typically maintained under controlled breeding and management conditions, with reduced exposure to environmental microbial sources, which may influence the composition and diversity of their associated microbiota.

Comparative analysis of *Escherichia coli* populations derived from urban and exhibition pigeons provides an opportunity to compare the distribution of selected virulence-associated genes between pigeon populations living under different environmental and management conditions [22,23,24]. Urban pigeons are exposed to heterogeneous ecological conditions, whereas exhibition pigeons are maintained under more controlled hygienic conditions. These differences provide the ecological context for comparing potential variations in the virulence gene repertoire and co-occurrence patterns of associated bacterial strains [9,10,11,13,25,26].

Understanding the distribution and co-occurrence of virulence-associated genes in pigeon-derived *Escherichia coli* isolates may provide baseline information on the diversity of these determinants in avian-associated bacterial populations. Such investigations may contribute to future comparative studies addressing potential connections among animal, environmental, and human-associated *E. coli* populations within a One Health framework [1,10,11,12,13,25,26].

The present study aimed to investigate the prevalence, distribution, and co-occurrence patterns of selected virulence-associated genes in *Escherichia coli* isolates recovered from urban and exhibition pigeons and to compare their virulence gene profiles between the two pigeon populations.

## 2. Materials and Methods

### 2.1. Sampling and Bacterial Isolates

A total of 453 faecal samples were collected from pigeons, including 153 samples from urban (synanthropic) pigeons and 300 samples from exhibition pigeons. From these samples, 133 *Escherichia coli* isolates were obtained (65 from urban pigeons and 68 from exhibition pigeons). Sampling was conducted between January 2023 and September 2024 in Timișoara, Romania. Urban pigeon samples were collected opportunistically from multiple public locations distributed throughout the city of Timișoara where free-living pigeon populations were regularly observed. Exhibition pigeon samples were obtained from pigeons belonging to different private breeders participating voluntarily in the study. Overall, sampling followed a convenience-based approach, with samples collected according to accessibility and voluntary participation. The exact number of urban sampling locations and participating exhibition-pigeon breeders was not systematically recorded during sample collection and therefore could not be retrospectively determined.

Urban pigeons were sampled from free-living populations in urban environments, whereas exhibition pigeons were sampled from birds maintained under controlled breeding and housing conditions. Neither urban nor exhibition pigeons were captured or handled during sampling. Freshly voided faecal samples were collected using sterile swabs. All birds were clinically healthy at the time of sampling.

Faecal samples were collected using sterile swabs, transported at 4 °C, and processed within 24 h. Samples were cultured on MacConkey agar and incubated aerobically at 37 °C for 18–24 h.

Samples from which colonies displaying the phenotypic and biochemical characteristics required for presumptive *E. coli* identification were not recovered were considered culture-negative for the purposes of the present study. Overall, *E. coli* was recovered from 133 of 453 faecal samples, corresponding to an isolation rate of 29.4%.

Presumptive *Escherichia coli* colonies were identified based on colony morphology, Gram staining, oxidase negativity, and standard biochemical characteristics, including indole production, lactose fermentation, citrate utilisation, and triple sugar iron (TSI) agar reactions, according to established microbiological protocols [27]. One representative colony displaying typical *E. coli* morphology was selected from each positive sample to avoid the duplicate inclusion of isolates originating from the same faecal sample. Pure cultures were obtained by subculturing and stored at −20 °C until further analysis.

### 2.2. Confirmatory Identification of Escherichia coli Isolates

To validate the conventional microbiological identification, a representative subset of 40 isolates (30.1% of the total collection) was independently confirmed by species-specific PCR using the TEcol553/TEcol754 primer pair, which amplifies a 258 bp fragment specific for the *Escherichia coli*/*Shigella* group. Positive amplicons were purified and subjected to bidirectional Sanger sequencing (Macrogen Europe B.V., Amsterdam, The Netherlands). The obtained sequences were compared with reference sequences available in the GenBank database using the BLAST algorithm. All analysed isolates were confirmed as *Escherichia coli*, supporting the reliability of the conventional microbiological identification procedure used for the entire isolate collection. This confirmatory analysis was performed prior to virulence gene profiling.

### 2.3. DNA Extraction

Genomic DNA was extracted from overnight bacterial cultures grown on nutrient agar plates. A single colony was suspended in sterile distilled water and subjected to thermal lysis at 95 °C for 10 min, followed by centrifugation to remove cellular debris.

The resulting supernatant containing genomic DNA was used as a template for PCR amplification. DNA samples were stored at −20 °C until further analysis. DNA concentration and purity were not quantified due to the use of crude lysates, which were directly used for PCR amplification.

### 2.4. Detection of Virulence Genes by PCR

The presence of six virulence-associated genes (*fimC*, *ompA*, *tsh*, *hlyA*, *astA*, and *iroN*) was investigated by polymerase chain reaction (PCR).

PCR reactions were performed in a final volume of 25 µL containing 2× PCR Master Mix (Thermo Fisher Scientific, Waltham, MA, USA), 0.5 µM of each primer, 2 µL of DNA template, and nuclease-free water.

Amplification conditions consisted of an initial denaturation at 95 °C for 5 min, followed by 35 cycles of denaturation at 94 °C for 30 s, annealing at 55–60 °C depending on the primer set for 30 s, and extension at 72 °C for 45 s, with a final extension at 72 °C for 5 min.

Gene-specific positive controls were included in all virulence gene PCR assays. *Escherichia coli* C749-89 (Statens Serum Institut, Copenhagen, Denmark) was used as a positive control for *fimC*, *hlyA*, and *astA*, whereas *E. coli* EF135 (Statens Serum Institut, Copenhagen, Denmark) was used as a positive control for *ompA*, *tsh*, and *iroN*. Nuclease-free water was included as a no-template control in each PCR run to monitor potential contamination.

Technical replicates were not systematically recorded for all PCR assays. Standard precautions were applied throughout the PCR workflow to minimise the risk of cross-contamination, including separation of pre- and post-amplification procedures and careful handling of samples and PCR products. PCR runs were considered valid only when the positive controls yielded the expected amplification products and no amplification was observed in the no-template control.

PCR products were separated by electrophoresis on 1.5% agarose gels stained with GelRed (Biotium Inc., Fremont, CA, USA) and visualized under UV light. A 100 bp DNA ladder was used as a molecular size marker.

Primer sets were selected from previously validated protocols and applied under published amplification conditions. A representative subset of isolates was independently confirmed by species-specific PCR followed by Sanger sequencing and sequence analysis using the NCBI Basic Local Alignment Search Tool (BLAST; National Center for Biotechnology Information, Bethesda, MD, USA), supporting the reliability of isolate identification prior to virulence gene screening.

Primers, annealing temperatures, and amplicon sizes are presented in Table 1. Primer sets were selected based on previously published studies.

Primers were selected from previously published and validated assays based on their reported specificity for the investigated virulence-associated genes and their previous application in avian and extraintestinal *E. coli* studies.

### 2.5. Virulence Gene Profile Analysis

The presence or absence of each virulence gene was recorded for all isolates, and a binary matrix was constructed. Based on these data, virulence gene profiles were defined according to the combinations of detected genes within each isolate.

The frequency of each virulence gene was calculated as the proportion of positive isolates among the total number of strains.

### 2.6. Co-Occurrence Analysis of Virulence Genes

Co-occurrence analysis was performed to evaluate the simultaneous presence of virulence genes within the same isolates. Pairwise combinations of genes were quantified, and the frequency of co-occurrence was calculated. Co-occurrence values were defined as the number of isolates in which each pair of genes was simultaneously detected.

These associations were used to construct a co-occurrence network in which nodes represented individual genes and edges indicated the frequency of their co-detection.

### 2.7. Heatmap Analysis

A heatmap was generated based on pairwise co-occurrence counts derived from the binary presence–absence matrix of virulence genes. For visualisation purposes, values were normalised using min–max scaling, while raw counts were retained as annotations. This approach allowed the visualization of co-occurrence patterns and associations among virulence genes.

### 2.8. Virulence Gene Score Analysis

A virulence gene score was calculated for each isolate as the total number of detected virulence-associated genes. Isolates were grouped according to the number of virulence-associated genes detected, allowing descriptive comparison of the complexity of the investigated gene profiles.

### 2.9. Association Matrix of Virulence Genes

An association matrix was constructed to assess relationships between virulence genes. Pairwise correlations were calculated based on presence–absence data to identify potential positive or negative associations among virulence determinants. Pairwise associations between virulence genes were quantified using the phi coefficient (φ), calculated from binary presence–absence data, with values ranging from −1 (negative association) to +1 (positive association). Because individual birds were not uniquely identified and the exact sampling location or breeder was not systematically recorded for each sample, potential clustering by location or breeder and repeated sampling could not be formally assessed. Statistical analyses therefore treated individual isolates as separate observations. This limitation should be considered when interpreting the statistical comparisons.

### 2.10. Statistical Analysis

Statistical analysis was performed to evaluate the distribution and associations of virulence genes.

The prevalence of each virulence gene was expressed as a percentage of positive isolates. Associations between virulence genes were analyzed using the Chi-square test (χ^2^). When expected frequencies were below five, Fisher’s exact test was applied.

Differences in the distribution of virulence genes between isolates obtained from urban and exhibition pigeons were also evaluated using the same statistical approaches.

All statistical analyses were conducted using R statistical software (version 4.3.1). Graphical representations, including bar plots and heatmaps, were generated using the ggplot2 package (version 3.5.0). The phi coefficient calculations were performed using custom scripts in R. A *p*-value < 0.05 was considered statistically significant. Prevalence estimates were calculated with 95% confidence intervals using the Wilson score method. Because the statistical analyses were exploratory, no correction for multiple comparisons (e.g., Bonferroni or false discovery rate adjustment) was applied. Therefore, the results of pairwise analyses should be interpreted with appropriate caution.

## 3. Results

### 3.1. Distribution of Virulence Genes

A representative subset of isolates was confirmed by species-specific PCR followed by Sanger sequencing and BLAST analysis, confirming their identification as *Escherichia coli*.

A total of 133 *Escherichia coli* isolates obtained from pigeon faecal samples (65 urban and 68 exhibition pigeons) were analysed for the presence of six virulence-associated genes. Among the investigated genes, *ompA* was the most frequently detected, being present in 117 isolates (88.0%; 95% CI: 81.4–92.5%), followed by *fimC*, identified in 88 isolates (66.2%; 95% CI: 57.8–73.7%). The iron acquisition-associated gene *iroN* was detected in 43 isolates (32.3%; 95% CI: 24.9–40.7%). Lower prevalence rates were observed for *tsh* (36/133; 27.1%; 95% CI: 20.2–35.2%) and *astA* (27/133; 20.3%; 95% CI: 14.3–28.0%), while *hlyA* was detected in 5 isolates (3.8%; 95% CI: 1.6–8.5%).

**Figure 1:**
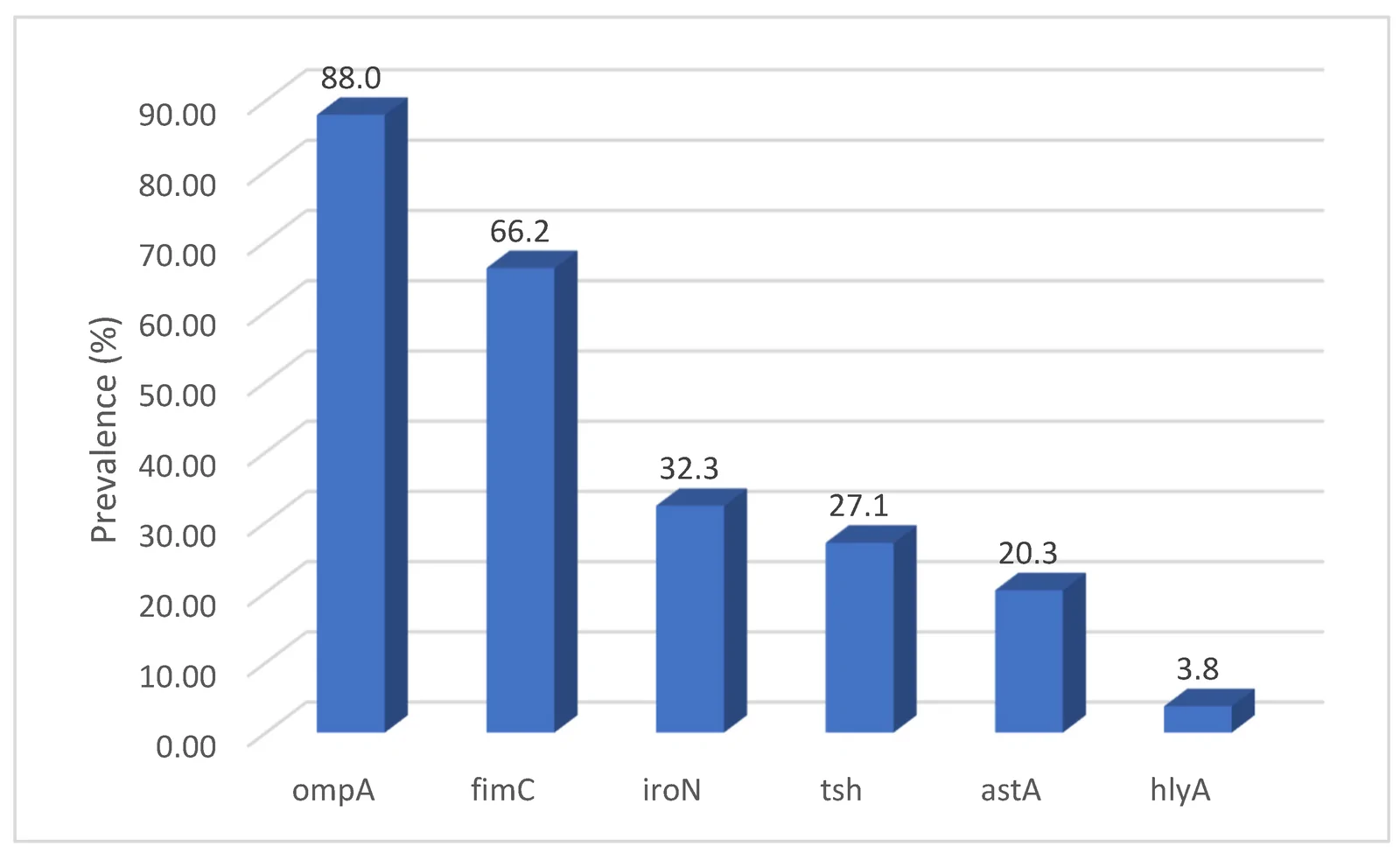
Prevalence of virulence-associated genes among the 133 *Escherichia coli* isolates. The frequency (%) of the investigated virulence genes (*fimC*, *ompA*, *tsh*, *hlyA*, *astA*, and *iroN)* is shown.

Overall, *fimC* and *ompA* were the most frequently detected virulence-associated genes. The distribution of virulence genes is presented in Figure 1.

### 3.2. Virulence Gene Profiles

Analysis of virulence gene combinations revealed a diverse distribution of profiles among the 133 isolates. The most common profile was *fimC + ompA*, identified in 33 isolates (24.8%), followed by isolates carrying only *ompA* (27 isolates, 20.3%).

Other frequently observed profiles included *fimC + ompA* + *iroN* (18 isolates, 13.5%), *fimC + ompA* + *tsh* (14 isolates, 10.5%), *ompA* + *iroN* (11 isolates, 8.3%), *fimC* + *astA* (9 isolates, 6.8%), and *tsh* alone (7 isolates, 5.3%).

**Figure 2:**
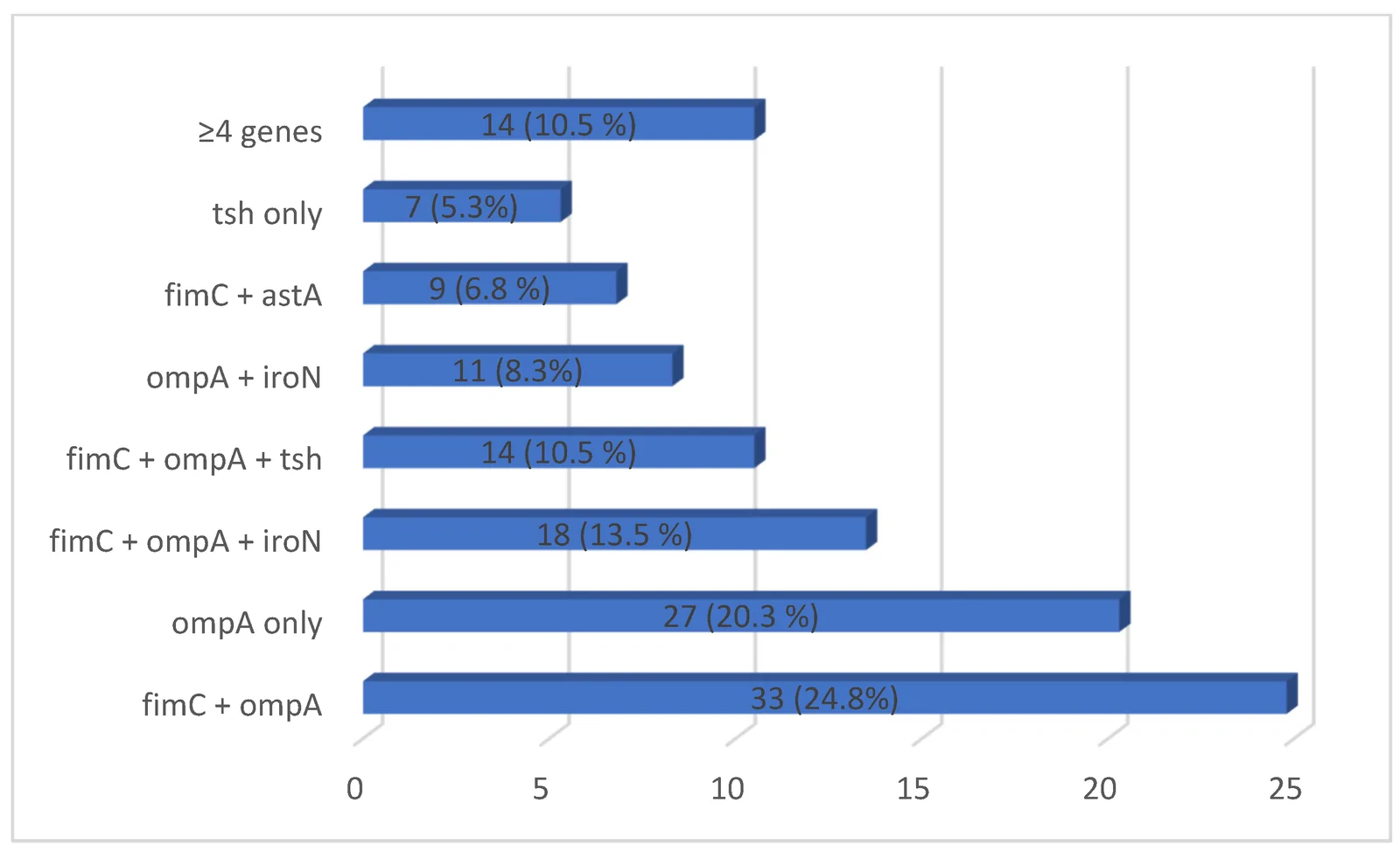
Distribution of the most frequent virulence gene profiles among the 133 *Escherichia coli* isolates. Bars represent the absolute number of isolates, with the corresponding percentage shown in parentheses.

Additionally, 14 isolates (10.5%) harbored four or more virulence genes, indicating the presence of more complex virulence profiles. The distribution of virulence gene profiles is shown in Figure 2.

### 3.3. Co-Occurrence of Virulence Genes

**Figure 3:**
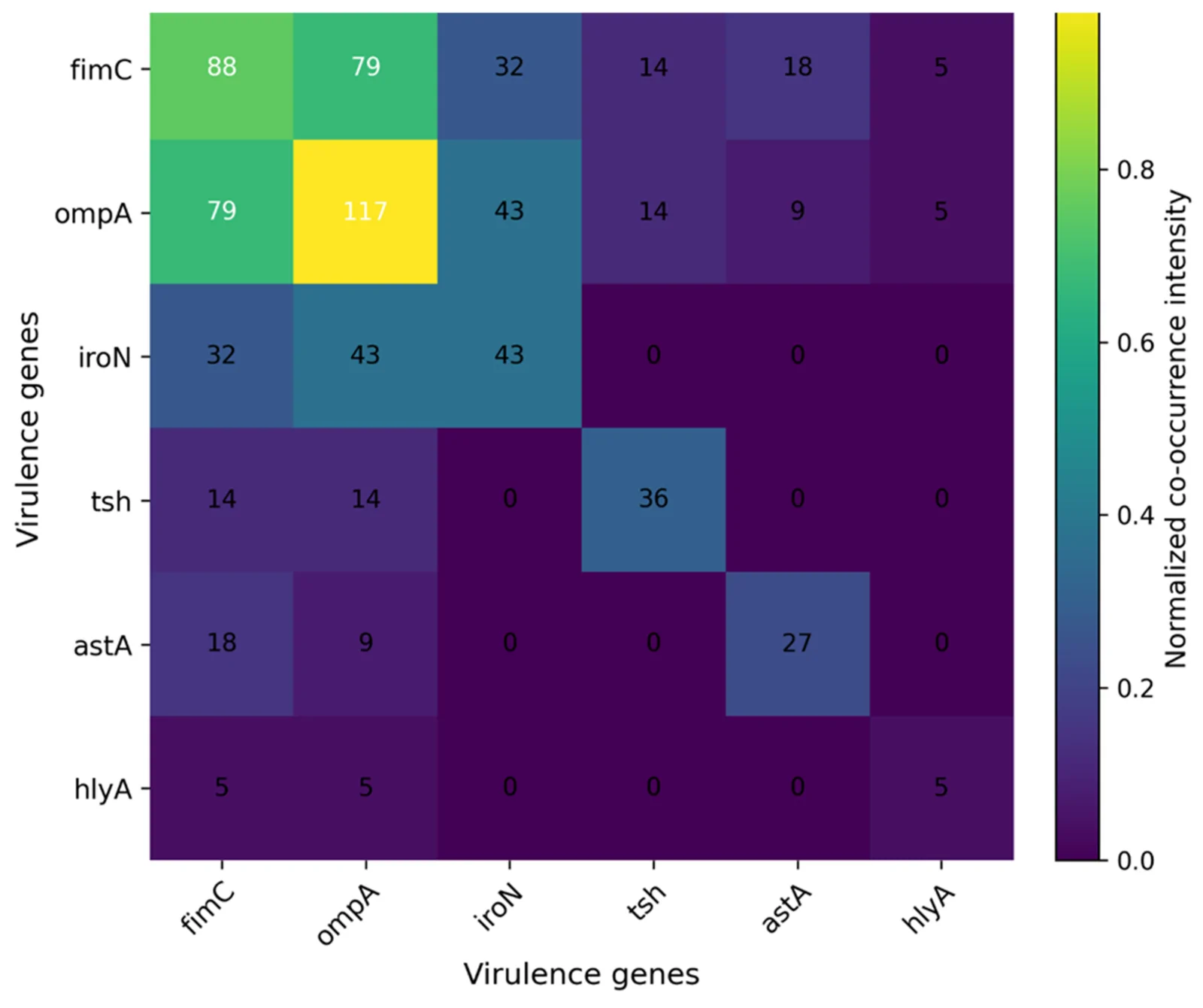
Heatmap illustrating the co-occurrence frequencies of the investigated virulence-associated genes. Off-diagonal values represent the number of isolates in which two genes were detected simultaneously, whereas diagonal values indicate the total number of isolates positive for each individual gene and do not represent gene co-occurrence.

Co-occurrence analysis revealed that several virulence genes were frequently detected together within the same isolates. Co-occurrence patterns among virulence genes are presented in Figure 3. The most frequently observed co-occurrence pattern was between *fimC* and *ompA*, which were simultaneously detected in 79 isolates (59.4%). However, the association between these genes was weak and not statistically significant (φ = 0.078, χ^2^ = 0.799, df = 1, *p* = 0.371). Therefore, the high frequency of co-detection primarily reflects the high individual prevalence of these genes rather than a strong statistical association.

Additional associations were identified between *ompA* and *iroN*, as well as between *fimC* and *iroN*, representing additional patterns of gene co-detection. These gene pairs showed less pronounced co-occurrence patterns than the most frequently observed *fimC–ompA* combination. Combinations involving *fimC*, *ompA*, and *tsh* were also observed, although at lower frequencies.

In contrast, genes such as *hlyA* and *astA* exhibited limited co-occurrence with other virulence determinants, reflecting their low prevalence among the analysed isolates. These genes were less frequently co-detected with the other investigated virulence-associated genes.

Overall, the observed co-occurrence patterns indicate that certain virulence-associated genes were frequently detected together within the analysed isolates. These patterns are illustrated in the heatmap shown in Figure 3. The heatmap and network analyses provide a descriptive overview of gene co-detection but do not imply functional linkage, co-regulation, or coordinated biological activity among the detected genes.

Cell colors represent min–max normalized co-occurrence intensity for visualization purposes, while the displayed values indicate the raw number of isolates in which each gene pair was co-detected.

### 3.4. Virulence Score Distribution

The analysis of virulence gene scores showed variation in the number of detected virulence-associated genes among isolates. The majority of isolates harbored two or three virulence determinants, whereas a smaller proportion carried only a single gene or more complex combinations involving four or more genes.

Isolates with higher virulence gene scores more frequently carried the adhesion-associated genes *fimC* and *ompA* together with the iron acquisition-associated gene *iroN*, consistent with the co-occurrence patterns observed in the present study. This pattern reflects the simultaneous detection of genes associated with adhesion and iron acquisition within the same isolates.

In contrast, isolates with lower virulence gene scores were typically characterized by the presence of single genes or limited combinations, often lacking iron acquisition or toxin-associated determinants. Overall, the distribution of virulence scores indicated a heterogeneous population, with a subset of isolates carrying more complex virulence gene profiles. However, PCR detection alone demonstrates only the presence of these genetic determinants and does not provide evidence of gene expression, protein production, or pathogenic phenotype.

### 3.5. Comparison Between Urban and Exhibition Pigeons

Comparison between isolates derived from urban (*n* = 65) and exhibition pigeons (*n* = 68) showed minor variations in the distribution of virulence-associated genes. The distribution of individual virulence genes in the two groups is presented in Table 2.

Overall, the prevalence of major virulence genes such as *ompA* and *fimC* was high in both groups, with only minor variations between urban and exhibition pigeons. Descriptively, isolates obtained from urban pigeons showed a somewhat broader range of virulence gene profiles; however, this observation should be interpreted cautiously because profile diversity was not formally compared using a specific diversity metric.

Similarly, no statistically significant differences were observed in the prevalence of the investigated virulence genes between the two groups (Table 2). Therefore, the present data do not provide statistical evidence of differences in the distribution of these genes between urban and exhibition pigeon isolates.

### 3.6. Heatmap Analysis of Virulence Gene Distribution

Heatmap visualisation of the presence–absence matrix revealed heterogeneous distribution patterns of virulence genes among the isolates. Distinct clusters of isolates sharing similar virulence gene profiles were identified, providing a descriptive representation of similarities in the detected gene profiles.

Adhesion-associated genes (*fimC* and *ompA*) were consistently present across the majority of isolates, forming the dominant pattern observed in the heatmap. Co-detection with the iron acquisition-associated gene *iroN* was also observed in a subset of isolates.

In contrast, peripheral genes such as *hlyA* and *astA* were detected in smaller subsets of isolates, reflecting their lower prevalence within the studied population. Overall, the heatmap illustrates the heterogeneous distribution of the investigated virulence-associated genes among the analysed isolates.

### 3.7. Network Analysis of Virulence Gene Co-Occurrence

Co-occurrence network analysis provided a descriptive visualisation of the frequency with which the investigated virulence-associated genes were detected together within individual isolates. The network showed that *fimC* and *ompA* had the highest degree of co-detection and were also frequently detected together with *iroN*. These patterns were consistent with the co-occurrence frequencies identified in the previous analysis.

In contrast, *tsh*, *astA*, and *hlyA* showed fewer co-occurrence relationships, consistent with their lower prevalence in the analysed population. The network represents patterns of gene co-detection only and should not be interpreted as evidence of functional linkage, co-regulation, genomic proximity, or coordinated contribution to pathogenicity.

## 4. Discussion

The present study investigated the distribution and co-occurrence of selected virulence-associated genes in *Escherichia coli* isolates obtained from urban and exhibition pigeons, providing insights into the structure of virulence gene repertoires in avian-associated strains. The results showed a predominance of adhesion-associated genes, accompanied by a moderate prevalence of the iron acquisition-associated gene *iroN* and a lower prevalence of the investigated toxin-associated genes [34,35].

Among the investigated virulence-associated genes, *ompA* and *fimC* were the most frequently detected, indicating that adhesion-associated determinants predominated in the analysed isolates. These findings are consistent with previous studies reporting a high prevalence of adhesion-associated genes in avian *Escherichia coli* isolates, supporting their role in host colonisation and persistence [36,37].

The detection of *iroN* in a substantial proportion of isolates is consistent with the recognised importance of iron acquisition systems in bacterial survival, particularly under iron-limited host conditions [7,8,38].

Iron is a critical factor for bacterial growth, particularly within host environments where its availability is limited. The co-detection of *iroN* with adhesion-associated genes such as *fimC* and *ompA* represents a recurrent pattern within the analysed isolates [38,39,40]. However, PCR-based co-detection alone does not demonstrate functional interactions between these determinants.

In contrast, the toxin-associated genes *hlyA* and *astA* were detected at lower frequencies. Similar observations have been reported in studies investigating virulence-associated genes in *E. coli* isolates from avian sources. Their lower prevalence and less frequent co-detection with other investigated genes indicate that these determinants were less represented within the gene panel analysed in the present study.

The analysis of virulence gene profiles revealed a diverse distribution of gene combinations, with a predominance of profiles involving adhesion determinants. The identification of more complex virulence gene profiles indicates greater diversity in the combinations of the investigated genes. However, because only a limited number of virulence-associated genes were analysed, these findings should not be interpreted as evidence of increased pathogenicity or as a basis for ExPEC/APEC classification. This variability in gene profiles is consistent with the heterogeneous distribution of the investigated virulence-associated genes and with the clustering patterns observed in the heatmap analysis [9,12,13,41].

Co-occurrence analysis provided additional insights into the structural organization of virulence genes. The frequent co-occurrence of *fimC* and *ompA* represents the predominant co-occurrence pattern identified in the analysed isolates. Similarly, the frequent association of *iroN* with adhesion-related genes reflects the co-occurrence patterns observed in the present dataset. In contrast, the lower prevalence and less frequent co-detection of *hlyA* and *astA* indicate that these genes were less represented within the investigated gene panel.

No statistically significant differences were observed in the prevalence of the investigated virulence-associated genes between isolates obtained from urban and exhibition pigeons (Table 2). Descriptively, urban isolates showed a somewhat broader range of virulence gene profiles and a slightly higher proportion of complex gene combinations, consistent with observations reported in previous studies of free-living pigeons [15,18]. Although these findings may suggest a possible trend, the absence of statistically significant differences does not support conclusions regarding the influence of environmental conditions on the distribution of virulence-associated genes. Therefore, the observed differences should be interpreted with caution and considered hypothesis-generating rather than confirmatory. Future studies including larger sample sizes, additional virulence markers, and genomic analyses will be required to determine whether these observations reflect true biological differences between urban and exhibition pigeon populations.

The detection of selected virulence-associated genes in pigeon-derived *Escherichia coli* isolates indicates that these bacteria carry virulence-associated determinants that warrant further investigation. However, the present study did not include comparative isolates from humans, domestic animals, or environmental sources, nor were genomic analyses performed. Therefore, the current findings should not be interpreted as evidence of zoonotic transmission, interspecies dissemination, or direct public health risk. Instead, they provide baseline data for future comparative and genomic studies aimed at better understanding the epidemiological relevance of pigeon-associated *E. coli* [37,38].

Antimicrobial susceptibility and antimicrobial resistance determinants were not investigated in the present study because they were outside its scope. Future studies integrating antimicrobial resistance profiling together with genomic analyses will provide a more comprehensive characterization of pigeon-derived *Escherichia coli* isolates.

This study has several limitations. First, the analysis was restricted to six selected virulence-associated genes and was not designed to establish ExPEC/APEC classification or to characterize the complete virulome of the investigated isolates. Additional virulence markers and whole-genome sequencing would provide a more comprehensive understanding of the genetic background and epidemiological relationships of these isolates. Furthermore, the study did not include comparative isolates from humans, domestic animals, or environmental sources, and no genomic relatedness analyses were performed. Therefore, no conclusions regarding zoonotic transmission or interspecies dissemination can be drawn from the present data. In addition, the functional expression of the detected genes was not assessed; therefore, their direct contribution to pathogenicity remains unknown. Moreover, DNA concentration and purity were not quantified because crude thermal lysates were used directly as PCR templates; therefore, variability in template quality may have influenced amplification efficiency. Finally, the use of a single isolate per sample may have limited the representation of within-sample diversity. The exact number of urban sampling locations and participating exhibition-pigeon breeders was not systematically recorded, and individual birds were not uniquely identified. Consequently, potential clustering by sampling location or breeder and the possibility of repeated sampling could not be formally evaluated. Statistical analyses therefore treated individual isolates as separate observations, which should be considered when interpreting comparisons between the two pigeon populations. No a priori sample-size or statistical power calculation was performed; therefore, the absence of statistically significant differences between the two pigeon populations should not be interpreted as evidence of equivalence or biological similarity. Furthermore, because the statistical analyses were exploratory, no correction for multiple comparisons was applied, which may increase the probability of type I error in pairwise analyses. Future studies integrating whole-genome sequencing, functional assays, and comparative genomic analyses will provide a more comprehensive characterisation of the epidemiological relevance of pigeon-derived *Escherichia coli* isolates.

## 5. Conclusions

The present study showed that pigeon-derived *Escherichia coli* isolates harboured diverse combinations of selected virulence-associated genes, with *fimC* and *ompA* being the most frequently detected determinants. The frequent co-occurrence of these genes with the iron acquisition-associated gene *iroN* was consistent with the co-occurrence patterns observed in the analysed isolates.

The observed diversity of virulence gene profiles, including the presence of more complex combinations in a subset of isolates, highlights heterogeneity in the distribution of the investigated virulence-associated genes among the analysed isolates. Co-occurrence, heatmap, and network analyses identified recurrent patterns of gene co-detection within the analysed isolates. These descriptive analyses provide baseline information for future genomic and functional studies.

No statistically significant differences were identified in the distribution of virulence-associated genes between isolates obtained from urban and exhibition pigeons. Descriptively, urban isolates showed a somewhat broader range of virulence gene profiles; however, this observation should be considered preliminary and requires confirmation in future studies.

Overall, pigeon-derived *Escherichia coli* isolates carried selected virulence-associated genes. These findings provide baseline information for future studies incorporating additional virulence markers, whole-genome sequencing, and comparative analyses to further characterise the epidemiological relevance of these isolates.

## Figures and Tables

**Table 1:** Primers used for detection of virulence-associated genes in *Escherichia coli* isolates.

Gene	Primer Sequence (5′–3′)	Amplicon Size (bp)	Annealing Temperature (°C)	Reference
*fimC*	F: GGGTAGAAAATGCCGATGGTG R: CGTCATTTTGGGGGTAAGTGC	477	55	[28]
*ompA*	F: AGCTATCGCGATTGCAGTG R: GGTGTTGCCAGTAACCGG	919	58	[29]
*iroN*	F: AAGTCAAAGCAGGGGTTGCCCG R: GACGCCGACATTAAGACGGA	665	57	[30]
*tsh*	F: ACTATTCTCTGCAGGAAGTC R: CTTCCGATGTTCTGAACGT	824	55	[31]
*hlyA*	F: AACAAGGATAAGCACTGTTCTGGCT R: ACCATATAAGCGGTCATTCCCGTCA	1177	58	[29]
*astA*	F: TGCCATCAACACAGTATATCC R: ACGGCTTTGTAGTCCTTCCAT	111	56	[32,33]

**Table 2:** Distribution of virulence genes in *Escherichia coli* isolates from urban and exhibition pigeons.

Gene	Urban Pigeons (*n* = 65)	Exhibition Pigeons (*n* = 68)	OR (95% CI)	*p*-Value
*ompA*	57 (87.7%)	60 (88.2%)	0.95 (0.33–2.70)	0.94
*fimC*	45 (69.2%)	43 (63.2%)	1.31 (0.64–2.69)	0.48
*iroN*	23 (35.4%)	20 (29.4%)	1.31 (0.63–2.72)	0.47
*tsh*	19 (29.2%)	17 (25.0%)	1.24 (0.58–2.67)	0.59
*astA*	15 (23.1%)	12 (17.6%)	1.40 (0.60–3.27)	0.44
*hlyA*	3 (4.6%)	2 (2.9%)	1.60 (0.26–9.88)	0.67

Values are expressed as *n* (%). OR, odds ratio; CI, confidence interval. Statistical comparisons between groups were performed using the Chi-square test or Fisher’s exact test, as appropriate. A *p*-value < 0.05 was considered statistically significant.

## Data Availability

All information is contained in the manuscript.
